# Primary colon adenocarcinoma with choriocarcinoma differentiation: a case report and review of the literature

**DOI:** 10.1186/s13256-020-02544-0

**Published:** 2020-11-16

**Authors:** Jessica Boyce, Karine Tawagi, John T. Cole

**Affiliations:** 1 Legacy Emanuel Medical Center, 2801 North Gantenbein Avenue, Portland, OR 97227 USA; 2 Ochsner Medical Center, 1514 Jefferson Highway, New Orleans, LA 70121 USA

**Keywords:** Choriocarcinoma, Colon, Adenocarcinoma, Choriocarcinoma metaplasia, Extragonadal choriocarcinoma, Tumor dedifferentiation, Nongestational choriocarcinoma

## Abstract

**Background:**

Choriocarcinoma is an aggressive malignancy of trophoblastic tissue, typically of gestational etiology. Sporadic, nongestational cases are rarely found outside of the gonads. There are only 31 cases of primary choriocarcinoma of the colon reported in the literature. As a consequence of their rarity and aggressive nature, timely diagnosis and effective treatment have proved challenging, and prognosis is very poor. For that reason, we present a rare case with prolonged survival in the youngest reported patient .

**Case presentation:**

A 26-year-old Caucasian woman presented with abdominal cramping and rectal and vaginal bleeding. Elevated serum human chorionic gonadotropin and an 8-cm right-sided mass seen on ultrasound suggested ectopic pregnancy. The patient was treated with methotrexate; however, her symptoms persisted, and her human chorionic gonadotropin levels continued to rise. Further workup showed a large mass of the sigmoid colon with multiple hepatic lesions suggestive of metastases. Preliminary pathology showed adenocarcinoma. Despite surgical resection and initiation of FOLFOX chemotherapy (folinic acid, fluorouracil, oxaliplatin), the patient had significant clinical deterioration, and her human chorionic gonadotropin increased exponentially. Further pathological review showed two distinct phenotypes: adenocarcinoma merging with choriocarcinoma. The result of evaluation of the metastatic lesions was also positive for choriocarcinoma. Treatment was promptly changed to a choriocarcinoma-targeting chemotherapy regimen of EMA/CO (etoposide, methotrexate, actinomycin D, cyclophosphamide, vincristine), resulting in rapid and dramatic response. The patient had mild progression after 1 year and was switched back to FOLFOX with bevacizumab. After five cycles, scans showed further progression, and the patient was started on third-line therapy with FOLFIRI (folinic acid, fluorouracil, irinotecan) and bevacizumab. Eighteen months after her diagnosis, the patient was alive and maintaining an overall response.

**Conclusions:**

Our patient achieved a marked response and prolonged survival. Although a comprehensive review of the literature showed that survival with these tumors has improved over the past 10 years, prognosis remains poor. Currently, there is no established algorithm for the management of these rare tumors, but both the literature and our patient’s case indicate that a choriocarcinoma-targeted regimen is critical for survival. Further evaluation of these rare tumors is warranted in order to identify pathological patterns that may help in the diagnosis, management, and survival of these malignancies.

## Background

Nongestational choriocarcinoma is a rare malignancy of trophoblastic tissue. These tumors most often arise in the uterus or gonads, but they have also been observed to arise from extragenital sites, typically in midline structures such as the retroperitoneum, mediastinum, and pineal gland. Even rarer is their occurrence in parenchymal organs such as the liver, lungs, or gastrointestinal tract. Within the gastrointestinal tract, these malignancies are most commonly found in the stomach. Extragonadal, nongestational choriocarcinomas of the bowel are extremely rare, with only 31 cases reported in literature [1–31]. Their diagnostic elusiveness, aggressive nature, and lack of established treatment management make prognosis for these malignancies extremely poor, with an average survival of only 8 months. We present a case of colon choriocarcinoma in the youngest documented patient whose treatment regimen resulted in marked response and longer-than-average survival.

## Case presentation

**Fig. 1:**
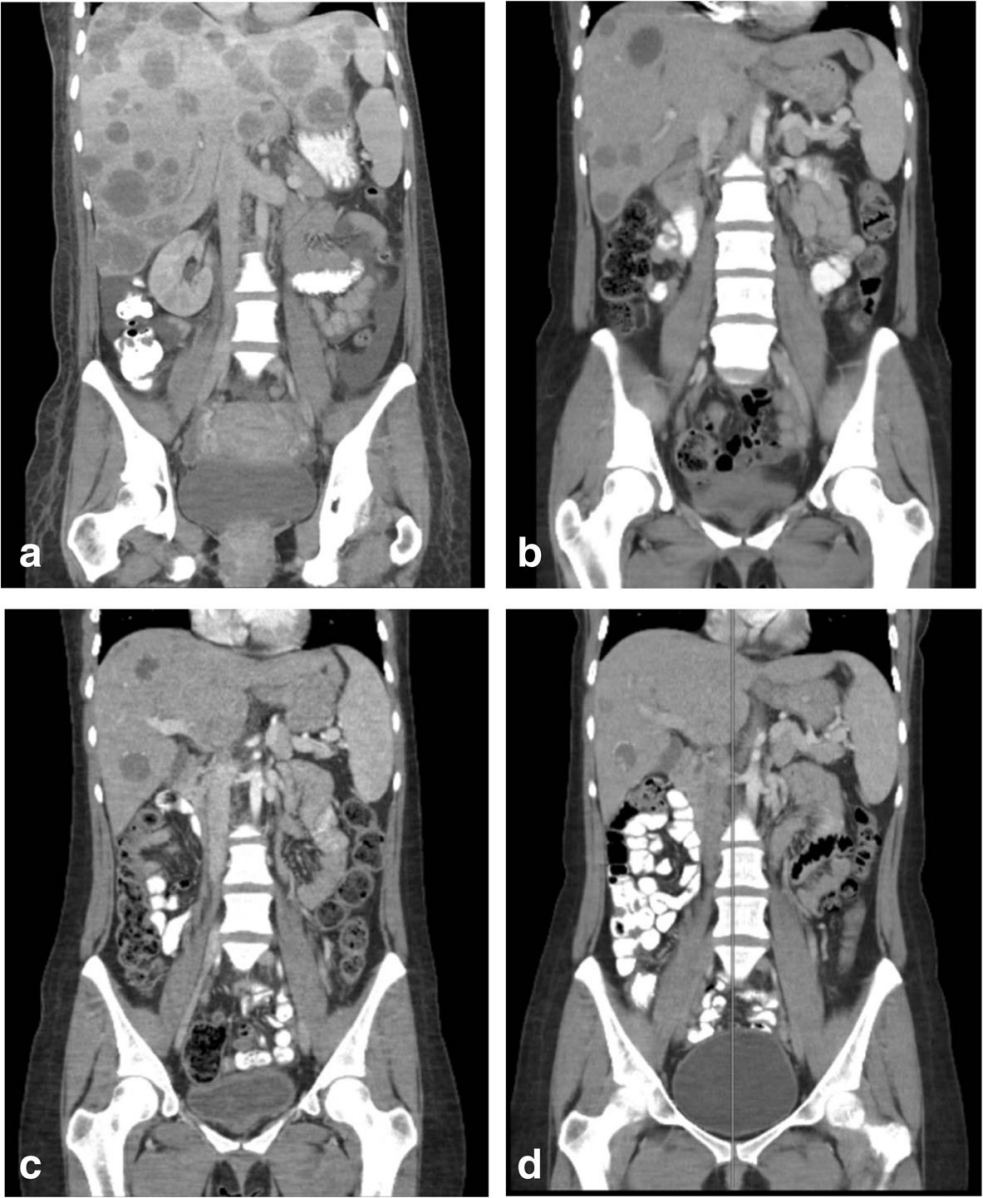
Radiographic evaluation of metastatic disease. **a** Baseline prior to initiating chemotherapy. **b** After five cycles of etoposide + methotrexate + actinomycin D ± cyclophosphamide + vincristine (EMA/CO) therapy. **c** After eight cycles of EMA/CO therapy. **d** After 11 cycles of EMA/CO therapy

A 26-year-old gravida 2, para 2 Caucasian woman presented with a 2-day history of bright red rectal and vaginal bleeding with abdominal cramping. Her reported last menstrual period was approximately 8 weeks prior. The patient’s history was notable for intermittent rectal bleeding for the past 2 years that had been attributed to postpartum hemorrhoids. Her serum human chorionic gonadotropin (hCG) was elevated at 1138 mIU/ml, and an in-office ultrasound revealed an 8-cm right-sided mass, strongly suggestive of an ectopic pregnancy. Diagnostic laparoscopy did not reveal an ectopic pregnancy; however, follow-up laboratory tests 1 week later showed a rise in β-hCG to 5511 mIU/ml. Repeat ultrasound showed an 8.9-mm mass but did not identify any intrauterine or extrauterine gestation. The patient was given a dose of methotrexate; however, she continued to experience intermittent lower abdominal cramping and vaginal bleeding. Follow-up laboratory tests 1 week later showed that her β-hCG had tripled to 16,326 mIU/ml. Repeat ultrasound was consistent with previous examinations showing a nonspecific heterogeneous right-sided mass but no gestational sac. The patient was given a second dose of methotrexate. She failed to follow up with repeat laboratory work as scheduled, and she continued to experience right lower quadrant pressure and intermittent vaginal bleeding. When she presented 15 days later, her repeat β-hCG had risen to 101,290 mIU/ml, at which time she was emergently admitted to the hospital. Computed tomography (CT) showed numerous hepatic masses measuring up to 2.3 cm scattered throughout the liver, strongly suggestive of widespread metastatic disease (Fig. 1a). An exploratory laparotomy revealed a sigmoid mass that almost completely obstructed the colon. A segmental resection of the sigmoid colon was performed with colostomy, as well as excisional biopsy of one of the hepatic nodules. Preliminary pathology showed high-grade adenocarcinoma of the colon.

The patient tolerated surgery well and was discharged to home to recover while arrangements for treatment were made, pending final pathological results. Contrary to expectations, the patient’s β-hCG continued to rise postoperatively. Two weeks after surgery, the patient returned to the hospital with new right upper quadrant and right chest wall pain with shortness of breath. Her β-hCG had markedly increased to 792,770 mIU/ml, and repeat CT showed new scattered pulmonary nodules and an interval increase in the size and number of hepatic lesions. Additional laboratory tests showed anemia, hyperbilirubinemia, and elevated lactate dehydrogenase. She was febrile and hypotensive and experienced episodes of tachycardia and dysrhythmia. The following day, updated pathological analysis noted invasive, high-grade colonic adenocarcinoma merging with a very poorly differentiated high-grade malignancy showing positivity for hCG, concerning for choriocarcinoma. Multiple medical colleagues, specialists, and pathologists were consulted, and specimens were sent to the Mayo Clinic for further analysis.

The patient continued to deteriorate clinically, with continued fevers, leukocytosis, and anemia, which were managed with antibiotics and transfusions. The results of blood cultures and a chest x-ray were negative, and the patient’s symptoms were ruled to be secondary to her malignancy. Given her rapidly worsening clinical picture and her tumor burden burgeoning in only 2 weeks, the decision was made to initiate FOLFOX therapy (folinic acid, fluorouracil, oxaliplatin). The patient received one dose and was discharged to home 1 week later. The following week, final pathology from the Mayo Clinic confirmed colon adenocarcinoma mixed with choriocarcinoma. Repeat laboratory tests showed continued increase in β-hCG to 916,335 mIU/ml. In light of the confirmed pathology and continued rise in β-hCG, the decision was made to modify therapy to a choriocarcinoma-targeted EMA/CO regimen (etoposide, methotrexate, actinomycin D, cyclophosphamide, vincristine) [32, 33].

Two weeks after initiation of EMA/CO, the patient’s β-hCG levels had dramatically dropped to 14,981 mIU/ml. After only five cycles, her β-hCG was 17 mIU/ml, and imaging showed a significant decrease in the size and number of her innumerable hepatic lesions (Fig. 1b), with almost complete resolution of her pulmonary disease. She continued to have marked radiographic response to EMA/CO therapy (Fig. 1b–d) and a continued decline in β-hCG, with the last quantitative measurement showing 2.7 mIU/ml after nine cycles.

**Fig. 2:**
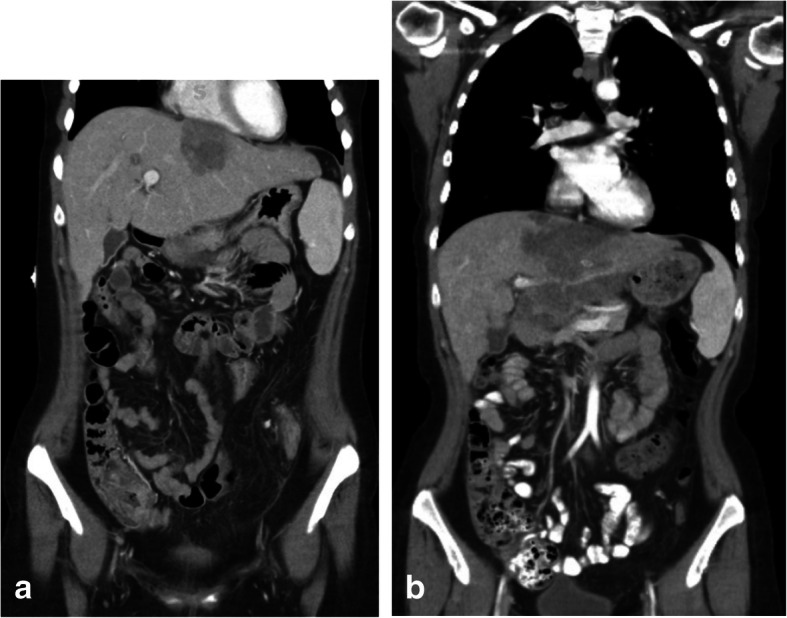
Radiographic evaluation of progression of disease. **a** Initial progression after 14 cycles of etoposide + methotrexate + actinomycin D ± cyclophosphamide + vincristine. **b** Further progression after five cycles of Folinic acid + fluorouracil + oxaliplatin + bevacizumab

The patient had completed a total of 14 cycles of EMA/CO when CT showed mild interval progression of some hepatic lesions (Fig. 2a). A core needle biopsy of one of the lesions showed metastatic adenocarcinoma consistent with colon cancer. The patient also began to experience abdominal pain, diarrhea, and fevers and was found to have gram-negative bacteremia. Following treatment with antibiotics, the patient was initiated on a FOLFOX + bevacizumab regimen. After five cycles of FOLFOX + bevacizumab, scans showed further progression of her lung lesions and mixed response of the liver lesions (Fig. 2b). Subsequently, her treatment regimen was changed to FOLFIRI (folinic acid, fluorouracil, irinotecan) + bevacizumab. The patient received one cycle of FOLFIRI + bevacizumab and was alive 18 months after her initial diagnosis.

## Pathology

**Fig. 3:**
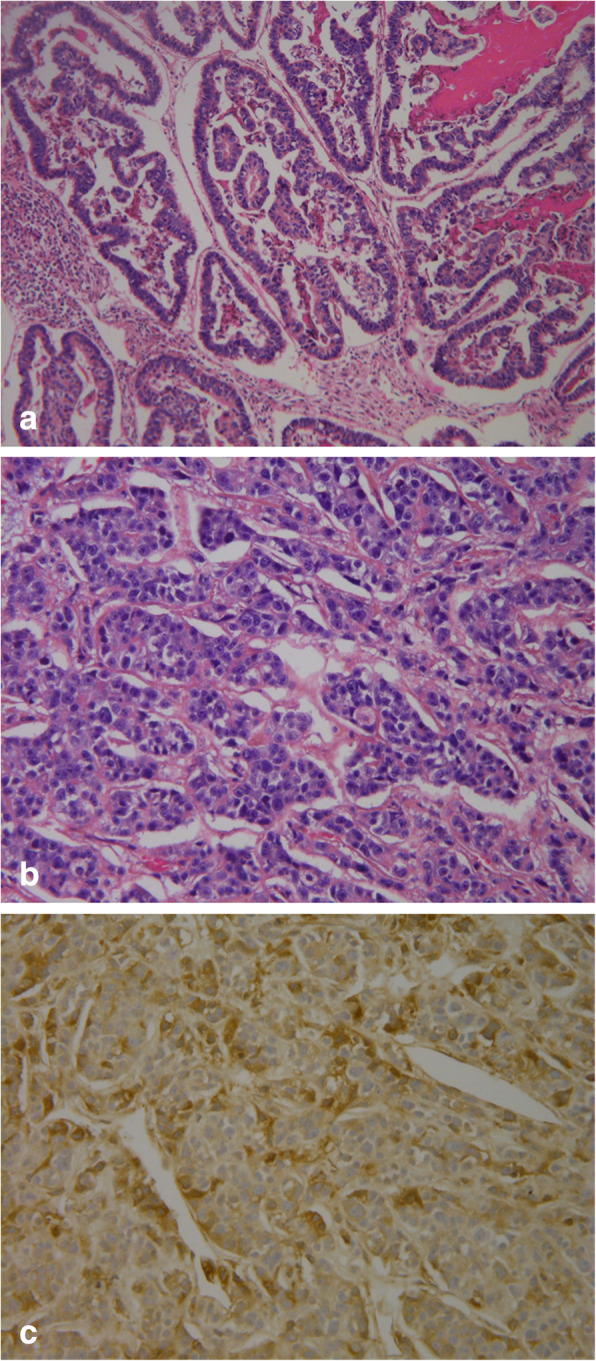
Pathology: histological and immunohistochemical evaluation. **a** Well-differentiated component. **b** Poorly differentiated component. **c** Positive human chorionic gonadotropin staining

This case was noted to be very difficult, requiring additional input from Mayo Medical Laboratories, where it was shared with multiple pathologists. Within the 18.5-cm segment of resected sigmoid colon submitted for analysis, a lesion measuring 8.5 × 3.8 × 3.0 cm was identified as invasive, high-grade colonic adenocarcinoma merging with a very poorly differentiated high-grade malignancy showing positivity for hCG (Fig. 3a–c). The lesion was found to arise within a preexisting tubulovillous adenoma with high-grade dysplasia and extended completely through the muscularis propria and serosal surfaces. The nodular lesion consisted of enlarged, cytologically malignant glands consisting of tall columnar cells exhibiting dysplastic nuclear features such as prominent enlargement, extensive stratification, nuclear hyaline, and total loss of polarity with large nucleoli and focal mitoses. These features became more prominent in the sheets of highly dysplastic cells seen in the intraserosal component of the tumor as it extended outward. Rare giant nuclei and occasional multinucleate cells were also identified with a somewhat complex cord growth pattern focally. There were also focal areas of necrosis, as well as slight cytoplasmic vacuolization observed within some cells. The serosal fat and overlying serosa both exhibited extensive hemorrhage. Additionally, three of five serosal lymph nodes exhibited metastatic high-grade carcinoma.

Analysis of the submitted liver specimen showed poorly differentiated carcinoma that was morphologically similar to the poorly differentiated component in the colon. Fragments showing extensive necrosis with recent hemorrhage were observed. Within the necrotic areas were irregular foci consisting of sheets of cohesive cells exhibiting highly pleomorphic nuclei with large nucleoli, irregular membranes, and loss of polarity. Some areas were noted to have larger cells with giant nuclei and occasional multinucleated cells.

Immunohistochemical stains were done on both the glandular and undifferentiated components of the colon malignancy, as well as the liver lesion (Table 1). The results echoed histopathological findings in that the malignancy progressively exhibited fewer features of adenocarcinoma and increasing features of choriocarcinoma as the tumor spread and metastasized.

**Table 1:** Immunohistochemical staining

	CEA	CDX2	hCG	CK7	CK20	PLAP
Colon lesion, glandular component	+++	+++	+++	–	–	–
Colon lesion, serosal component	+++	–	+++	–^a^	–^a^	
Liver lesion	–	–	+++	+++	–	+++

^a^Negative, with weak positivity in isolated cells only

*CDX2* caudal type homeobox-2, *CEA* carcinoembryonic antigen, *CK7* cytokeratin 7, *CK20* cytokeratin 20, *hCG* human chorionic gonadotropin, *PLAP* placental alkaline phosphatase

Genomic testing was significant for TP53-pR175H with a 72% estimated variant allele frequency. Microsatellite instability testing showed intact nuclear enzymes for MLH1, MLH2, MSH6, and PMS2 in all specimens. Tumor mutational burden was low (two mutations/megabase). Several targeted genomic alterations were tested, including *BRAF, KRAS, MYC, NRAS, ROS,* and *RET*. No mutations were identified.

Of note, there were multiple tubular and tubulovillous adenomas found to be present within uninvolved areas of the patient’s colonic mucosa. Certain architectural features were focally suggestive of malignancy and noted to be arguably classified as intramucosal carcinoma (versus high-grade dysplasia). For example, multiple polypoid fragments of tubulovillous colonic mucosa were seen to exhibit foci of dysplasia consisting of increased cellularity associated with prominent nuclear stratification, enlargement, loss of polarity, large centers, and nucleoli. Some of these foci were found to be cytologically malignant. Some of the glands exhibited a very complex cribriform and microglandular pattern and had frequent mitoses.

## Discussion

Choriocarcinoma is an aggressive, hCG-secreting tumor that rarely arises in extragenital tissues. Upon review of the literature (Table 2) [1–31], most colon choriocarcinomas arise in the sigmoid (*n* = 11) or ascending (*n* = 9) colon. They are highly invasive with early hematogenous metastatic spread. Of the reported cases, all but two had confirmed metastases, most commonly to the liver (*n* = 22) and/or lungs (*n* = 15). Consequently, patients with these tumors frequently present with signs of significant bleeding and often die of complications of liver or respiratory failure. Per the literature, the average age of presentation is 50.5 years, and the female-to-male ratio is 1.2 to 1.

**Table 2:** Review of colon choriocarcinoma cases

Case (*N* = 32)	Sex/age (years)	Primary tumor		Metastatic tumor		Peak serum β-hCG (mIU/ml)	Treatment/management	Survival (months)
		Location	Histology	Location	Histology			
Park and Reid 1980 [1]	F/49	Sigmoid	A + C	Liver, lung, diaphragm, rLN, dLN	C	NR	Palliative resection with colostomy	3.5
Nguyen 1982 [2]	M/74	Sigmoid	A + C	Liver	NR	186,000	Laparotomy with sigmoidectomy	2.5
Ordóñez and Luna 1984 [3]	F/35	Ascending/cecum	A + C	Liver, lung, pleura, pericardium, rLN, dLN, bone	C	1612	Explorative laparotomy with right hemicolectomy	3
Kubosawa *et al.* 1984 [4]	F/50	Sigmoid	A + C	Liver, lung, dLN	C	230,000	Hartmann operation with colostomy	1
Metz *et al.* 1985 [5]	F/42	Sigmoid	A + C	Liver, lung, spleen, rLN, dLN	A + C	154,000	Explorative laparotomy	1
Lind and Haghighi 1986 [6]	M/42	Ascending	C	Liver, lung, spleen, bone, dLN, kidneys, brain	C	610,000	Laparotomy Bleomycin + vinblastine + cisplatin Whole-brain irradiation	1
Ostör *et al.* 1993 [7]	F/28	Rectum	A + C	Liver, rLNs	C + YS	1.4 × 10^6^	Anterior resection EMA + leucovorin (one dose)	3
Rodilla *et al.* 1995 [8]	M/84	Rectum	C	rLN	C	5	Local radiotherapy Cyclophosphamide + doxorubicin (four cycles) Laparotomy with rectosigmoidectomy	3.5
Tokisue *et al.* 1996 [9]	F/29	Rectum	A + C	Lung, brain	NR	49,000	EMA Surgical resection EMA + cisplatin + doxorubicin (two cycles) Whole-brain irradiation	10
Kim *et al.* 1997 [10]	F/66	Ascending	A + C	NR	NR	NR	Surgical resection	NR
Oh *et al.* 1997 [11]	M/60	Sigmoid	A + C	Liver, lung, thyroid, brain, larynx, rLN	C	8772	Sigmoidectomy MTX + vincristine + VP16 + leucovorin (six cycles) Whole-body radiation	15
Kiran *et al.* 2001 [12]	M/68	Rectum	A + C	Liver, rLN	A + C	> 700,000	Hartmann procedure Total colectomy	NR
Petricek, 2001 [13]	M/29	Transverse	A + C + YS	Liver, rLN, dLN	YS	729 μg/L	None	6 days
Le *et al.* 2003 [14]	M/73	Ascending	C	Lung, brain, kidney, pancreas, rLN, descending colon	C	146,000	None	10 days
Verbeek *et al.* 2004 [15]	F/54	Rectum	A + C	Liver, lung	C	102,000	Surgical resection VIP-cisplatin (four cycles) Thoracotomy with resection of residual lung metastases	8
Jeong *et al.* 2007 [16]	M/52	Rectum	A + C	Liver, lung, rLN	C	42,910	Abdominoperineal resection BEP-cisplatin	1.5
Kawahara *et al.* 2009 [17]	M/62	Ascending/cecum	A + C + YS	Liver, intramural, dLN	C + YS	NR	Right hemicolectomy 5-FU via hepatic arterial infusion	4
Froylich *et al.* 2010 [18]	F/57	Descending	C	Lung, bone, brain	A + C	13,000	Left colectomy VIP-cisplatin + mesna (four cycles) Bilateral upper lobectomies	16
Gifford *et al.* 2010 [19]	F/33	Colon (unspecified)	C	Liver, lung, omentum	NR	116,725	Chemotherapy, unspecified (several cycles)	4.5
Harada *et al.* 2012 [20]	F/58	Sigmoid	A + C	None	–	2420	Hartmann operation Mitomycin C EMA UFT + leucovorin (ten cycles)	60+
Jiang *et al.* 2013 [21]	M/36	Ascending	C	Liver, rLN	C	10,000	Colectomy BEP-cisplatin	6+
Maehira *et al.* 2013 [22]	M/68	Sigmoid	A + C	Liver, rLN	C	3929 ng/ml	Sigmoidectomy mFOLFOX6 mFOLFOX6 + bevacizumab (eight cycles) FOLFIRI + bevacizumab	9
Mardi *et al.* 2014 [23]	F/54	Rectum	A + C	rLN	NR	4568	Radical resection 5-FU + leucovorin	50 days
Tsujimoto *et al.* 2014 [24]	M/38	Sigmoid	A + C	Liver, adrenals, peritoneum	NR	1.32 ng/ml	Sigmoidectomy mFOLFOX6 + bevacizumab	59 days
Julián *et al.* 2015 [25]	M/45	Sigmoid	A + C	Liver, rLN	A + C	22,288	BEP-cisplatin (two cycles) Exploratory laparotomy with palliative colostomy	3
Oh *et al.* 2015 [26]	F/61	Sigmoid	A + C	Liver	NR	35	FOLFIRI + bevacizumab (five cycles) FOLFOX (three cycles)	13
Koelzer *et al.* 2016 [27]	F/61	Cecum	C	Liver, peritoneum, spleen, uterus, ovary, rectum, bone	NR	70,173	Right hemicolectomy XELOXIRI + bevacizumab (two cycles) BEP Carboplatin + bleomycin Ifosfamide + vinblastine Surgical tumor debulking Paclitaxel (two cycles)	34
Parker *et al.* 2016 [28]	F/51	Ascending	A + C	Liver, mesentery, dLN	NR	96,000	EMA/CO	NR
Pezzuto *et al.* 2017 [29]	M/47	Cecum	C	Lung, brain	NR	NR	Colectomy with lymph node dissection	NR-”died in a little time”
Iliev *et al.* 2018 [30]	−−/54	Ascending	C	rLN	C	67.1	Extended right hemicolectomy EMA (3 months)	12+
Fang *et al.* 2019 [31]	F/29	Ascending	C	Lung, vertebrae	NR	NR	None	Few days
Our patient	F/26	Sigmoid	A + C	Liver, lung	A + C	916,335	Sigmoidectomy FOLFOX (one dose) EMA/CO (14 cycles) FOLFOX + bevacizumab (5 cycles) FOLFIRI + bevacizumab	TBD

*Abbreviations: A* adenocarcinoma, *BEP* bleomycin + etoposide (VP16) + platinum, *C* choriocarcinoma, *dLN* distant lymph nodes, *EMA/CO* etoposide + methotrexate (MTX) + actinomycin D ± cyclophosphamide + vincristine, *FOLFIRI* folinic acid + fluorouracil (5FU) + irinotecan, *FOLFOX* folinic acid + fluorouracil (5FU) + oxaliplatin, *NR* not reported, *rLN* regional lymph nodes, *TBD* to be determined, *UFT* uracil + tegafur, *VIP* etoposide (VP16) + ifosfamide + cisplatin, *XELOXIRI* capecitabine, oxaliplatin, irinotecan, *YS* yolk sac

Of the cases reported, primary colon lesions were predominantly biphasic tumors, comprised of adenocarcinoma and choriocarcinoma (*n* = 23); yet, 76% of metastases with reported histology were solely choriocarcinoma. In our patient’s case, there were features of choriocarcinoma and adenocarcinoma in both the primary and metastatic lesions. However, it was noted by pathology that the cellular architecture became poorer and less differentiated as the tumor extended and spread, with the metastatic liver lesion more closely resembling choriocarcinoma than the adenocarcinoma of its primary source. This histological pattern is consistent with other reported cases and congruent with Pick’s [34] widely accepted theory that these malignancies are the result of a primary adenocarcinoma undergoing retrodifferentiation, or trophoblastic metaplasia [35], resulting in transformation into progressively more primitive cellular forms until they are indistinguishable from choriocarcinoma. The process of retrodifferentiation, or dedifferentiation, can be clearly demonstrated in cases where the choriocarcinoma merges with the adenocarcinoma. As observed in our patient’s case, there is often a transitional zone [36] between the two tumor types that exhibits certain histologic features of both malignancies. Examination of these transitional forms allows the direct visualization of the dedifferentiation process, also referred to as “opisthoplasia,” occurring sequentially *in situ*. In cases where there is solely choriocarcinoma without any adenomatous component identified in the primary lesion, it is believed that trophoblastic metaplasia has occurred to the extent that the more aggressive choriocarcinoma has essentially overtaken the adenomatous tumor, resulting in complete tumor dedifferentiation [37].

Genetic instability is believed to be responsible, at least in part, for the dedifferentiation of adenocarcinoma and its divergence into multiple phenotypes [5]. Interestingly, it has been demonstrated that up to 43–52% of morphologically typical adenocarcinomas have positive immunohistochemistry for hCG [38, 39], suggesting some shared functional commonality. Upon reviewing the literature, only a few cases were determined to be pure primary choriocarcinoma of the colon without any adenomatous component. Of those, at least 30% were initially diagnosed as adenocarcinoma on the basis of histology but were ultimately ruled to be choriocarcinoma on the basis of immunohistochemical profiles showing strong positivity for hCG, with mixed responses for cytokeratin 7 (CK7), caudal type homeobox-2 (CDX-2), and villin, among other stains [21, 30, 31]. Taking into account the frequency with which otherwise normal colorectal adenocarcinomas display hCG positivity, it is reasonable to wonder whether these cases were actually pure choriocarcinomas or rather examples of genetic instability in action resulting in the divergence of adenocarcinoma cells into different subpopulations, including a transitional subgroup showing mixed histological and immunohistochemical features. Although they may have been pure choriocarcinomas as initially diagnosed, it is possible they are simply additional variations of the transitional zones described previously in the literature.

Despite a common functional component to choriocarcinoma, the dedifferentiation of colorectal adenocarcinoma to choriocarcinoma is extremely rare. Additionally, these particular carcinomas tend to occur in a younger population than other colon malignancies or even choriocarcinomas elsewhere in the gastrointestinal tract, raising the question if there could potentially be an underlying genetic component or hereditary cancer syndrome involved. To that point, the biopsy in this case noted multiple adenomas with malignant features in uninvolved tissue. However, genomic testing showed no microsatellite instability or gene alterations aside from p53. The patient had no personal or family history of gastrointestinal disease or malignancy. Furthermore, of all the reported cases of primary colon choriocarcinoma, there were only four cases with any prior history of gastrointestinal pathology. These included a single rectal polyp removed 10 years prior to diagnosis [9], a patient with diverticular disease who was 31 months postsurgical resection of an annular carcinoma of the rectum [12], a patient with Lynch syndrome who had gastric cancer treated 25 years ago [26], and a perineal abscess in a patient with Crohn disease [31]. There were two additional patients with a remote history of cancer: one was 2 years postlobectomy for squamous cell lung carcinoma [2], and the other was 3 years postmastectomy for breast cancer [20]. Ultimately, the lack of a strong oncological or gastrointestinal history or identifiable genomic mutational pattern in these patients attenuates the theory of a hereditary component in the tumorigenesis of this particular malignancy.

Identifying potential genetic drivers of these tumors, whether inherited or sporadic, should continue to be pursued because it could provide valuable insight regarding the detection and management of an aggressive malignancy with a very poor prognosis. The average survival from diagnosis is only ~ 8 months; however, it should be noted that this is longer than previously reported in the literature, possibly reflecting more effective treatment over the last 10 years. Although there is currently no consensus or standard treatment regimen for this rare malignancy, standard chemotherapy for gestational trophoblastic neoplasias such as EMA/CO, VIP (etoposide, ifosfamide, cisplatin), or BEP (bleomycin, etoposide, platinum) have most commonly been used. The literature suggests that the presence of choriocarcinoma has the greatest impact on prognosis, even more than delay in diagnosis, metastatic disease burden, or the coexistence of adenocarcinoma [14]. Review of cases with longer-than-average survival, including our patient’s case, shows that prompt initiation of chemotherapy directed at choriocarcinoma rather than adenocarcinoma is essential to survival.

However, it is important to recognize the heterogeneity of these tumors. Although the presence of choriocarcinoma is observed to be the predominant factor influencing prognosis, these tumors are noted to be more aggressive than traditional gestational trophoblastic neoplasias [33]. In our patient’s case, even with effective treatment with EMA/CO, the adenocarcinoma component of the tumor eventually progressed. It is suggested that the differences in cellular origin could be associated with differences in chemosensitivity in these tumors [22]. Consequently, the hybrid population of these tumors necessitates that management include multiagent therapy, ideally targeting both choriocarcinoma and adenocarcinoma [20].

## Conclusion

Our patient was a woman with primary choriocarcinoma of the sigmoid colon. She is the youngest reported patient with this malignancy and achieved longer-than-average survival secondary to radical surgical resection, a single dose of FOLFOX, prolonged EMA/CO therapy, and multiple cycles of FOLFOX with bevacizumab, followed by FOLFIRI with bevacizumab. The patient is maintaining her overall response and remained alive 18 months after her initial diagnosis.

Colorectal choriocarcinoma is an extremely rare malignancy with a very poor prognosis. It disproportionately affects younger patients and has no established standard treatment. Given that timely diagnosis has proved challenging and its aggressive course, one should maintain a high index of suspicion in younger patients presenting with symptoms of colon malignancy or in patients with elevated β-hCG for whom gestation cannot be confirmed. Further investigation of molecular, genetic, and immunohistochemical characteristics of these tumors is encouraged because identification may lead to more timely and accurate diagnoses, development of a standard treatment regimen, and prolonged survival in this unique group of patients.

## Data Availability

Not applicable.

## Abbreviations

BEP: Bleomycin + etoposide (VP16) + platinum
CDX-2: Caudal type homeobox-2
CEA: Carcinoembryonic antigen
CK7: Cytokeratin 7
CK20: Cytokeratin 20
CT: Computed tomography
EMA/CO: Etoposide + methotrexate (MTX) + actinomycin D ± cyclophosphamide + vincristine
FOLFIRI: Folinic acid + fluorouracil (5FU) + irinotecan
FOLFOX: Folinic acid + fluorouracil (5FU) + oxaliplatin
hCG: Human chorionic gonadotropin
PLAP: Placental alkaline phosphatase
UFT: Uracil + tegafur
VIP: Etoposide (VP16) + ifosfamide + cisplatin
XELOXIRI: Capecitabine, oxaliplatin, irinotecan
